# Effectiveness of rTMS compared to SSRI as early treatment of depression – study protocol of a randomized controlled trial (Early-TMS)

**DOI:** 10.1007/s00406-025-01975-4

**Published:** 2025-02-22

**Authors:** Julia Becker-Sadzio, Bettina Brendel, Simone Weller, Edmund Bornheimer, Ulrike Mehlig, Frank Padberg, Ulrike Vogelmann, Thomas Kammer, Wolfgang Strube, Peter Martus, Andreas J. Fallgatter, Christian Plewnia

**Affiliations:** 1 Department of Psychiatry and Psychotherapy, Neurophysiology & Interventional Neuropsychiatry, University Hospital Tübingen, Tübingen, Germany; 2 DZPG (German Center for Mental Health), Berlin, Germany; 3 Lived Experience Representative Early-TMS Study, Tübingen, Germany; 4 Department of Psychiatry and Psychotherapy, LMU University Hospital, Munich, Germany; 5 Department of Psychiatry and Psychotherapy, University Hospital, Technical University of Munich (TUM), Munich, Germany; 6 Section for Neurostimulation, Deptartment of Psychiatry and Psychotherapy III, Ulm University, Ulm, Germany; 7 Department of Psychiatry, Psychotherapy and Psychosomatics, Medical Faculty, University of Augsburg, Augsburg, Germany; 8 Institute for Clinical Epidemiology and Applied Biostatistics, University of Tübingen, Tübingen, Germany

**Keywords:** Repetitive transcranial magnetic stimulation (rTMS), Theta burst stimulation (TBS), Early intervention, Medication-naïve patients, Selective serotonin reuptake inhibitor (SSRI)

## Abstract

Psychotherapy and antidepressant medication are considered first-line treatment options for major depressive disorder (MDD). However, a high proportion of patients do not respond to initial treatment, underlining the need for alternative treatment methods. Repetitive transcranial magnetic stimulation (rTMS) has been established in the treatment of MDD, but the available evidence is limited to forms of MDD with varying degrees of treatment resistance. Randomized-controlled trials (RCT) investigating first-line treatment with rTMS in comparison with first-line antidepressant medication are warranted to further position rTMS within current treatment algorithms for MDD. In this two-stage, therapy response-adapted, randomized multi-center phase 2 rater blinded trial, 106 medication-naïve patients suffering from MDD will be enrolled. In Stage I, participants receive one of the two treatment options for four weeks: either daily bilateral theta burst stimulation (TBS), a patterned and time-saving form of rTMS, or antidepressant medication with selective serotonin reuptake inhibitors (SSRI). The allocation to Stage II occurs therapy response-adapted. Therefore, patients either receive maintenance treatment or will be switched to the respective other treatment arm. Primary outcome is the comparison between the two study arms with regard to therapy response measured by the Montgomery-Asberg Depression Rating Scale (MADRS) after 4 weeks at the end of Stage I. The aim of the study is to provide reliable first evidence and effect size measures of rTMS as first-line treatment compared to SSRI treatment. Positive results will help to implement rTMS in early stages of MDD. Trial registration: ClinicalTrials.gov ID: NCT06545474, August 15, 2024.

## Background

Major depression (MDD), a mental illness with rising prevalence [1], severely impairs quality of life, autonomy, social integration and life expectancy [2]. Psychotherapy and medication are effective treatments of MDD. Nevertheless, a large proportion of patients do not respond sufficiently to the initial treatment [3] and approximately 60% will experience a recurrent or chronic course of illness under available treatment options [4]. Psychotherapy and antidepressant medication are considered first-line treatment opportunities in MDD. The often-insufficient response to antidepressants combined with limited psychotherapeutic treatment resources lead to undertreatment for many MDD patients. Non-pharmacological treatment options such as non-invasive brain stimulation techniques might provide the opportunity to better treat MDD. In particular, repetitive transcranial magnetic stimulation (rTMS) has been established as an effective treatment for MDD [5]. Although rTMS is safe, has few side effects and shows good efficacy [5–7], its use is still limited to forms of MDD, which respond inadequately to antidepressant treatment. In these patients, rTMS is superior to pharmacological treatment strategies such as switching or augmentation even in treatment resistant depression (TRD) [8, 9]. Evidence for the benefit of rTMS in early phases of the disease compared to a first pharmacological treatment is still lacking and therefore limits the use of this well-tolerated treatment option in this large and often underserved patient group [10]. With theta burst stimulation (TBS), a significantly shorter and more cost-efficient variant of rTMS is available. Based on recent evidence for non-inferiority [6, 11, 12], TBS has largely replaced conventional 10 Hz rTMS as the standard rTMS protocol in the treatment of MDD. As for TBS however, available evidence refers almost exclusively to MDD forms with variant degrees of treatment resistance. According to the current approvals and guideline recommendations, rTMS is predominantly considered after several unsuccessful treatment attempts.

However, based on its efficacy, favourable side-effect-profile, and high patients‘ acceptance, its use as first-line treatment of MDD seems obvious and promising [13]. For this purpose, studies investigating rTMS in early phases of depression, and particularly compared to other first-line treatments such as selective serotonin reuptake inhibitors (SSRI) medication and psychotherapy, are needed.

To our knowledge, a comparison of the efficacy of TBS and antidepressant medication in medication-naïve MDD patients does not exist. The few available studies are limited to rTMS as add-on treatment to ongoing antidepressant medication [14–16]. The recommendation of rTMS as a first-line treatment requires a head to head comparison to antidepressant medication. Therefore, we investigate rTMS as first-line treatment in an early phase of MDD by focusing on patients who have not received adequate antidepressant treatment before. Since SSRIs are by far the most commonly prescribed antidepressants in primary care, and escitalopram is one of the most widely used and well tolerated substances in this group [17], this SSRI will be used as comparator. Regarding the rTMS treatment protocol, we will follow most recent evidence and our own line of research [18–20] by augmenting left-sided intermittent TBS (iTBS) [20] with right-sided continuous TBS (cTBS) [20].

Insufficient treatment response is a common challenge in early phases of MDD [21]. For this, clinical guidelines recommend to check treatment success of antidepressant therapy after 3–4 weeks and, in cases of insufficient response, to adapt the treatment strategy accordingly. If rTMS is meant to be considered as a feasible first-line intervention in MDD, the question of the optimal sequence of interventions arises. It is also interesting to examine the extent to which patients’ preferences, beliefs, and expectations of treatment efficacy influence its success [22].

With this study, we aim to fill this knowledge gap accordingly. *First*, we want to provide reliable evidence and effect size measures of TBS compared to SSRI as an early intervention for MDD in medication-naïve patients. *Second*, we aim to obtain initial data about the influence of intervention order on the effectiveness of treatment and feasibility of the two-stage response-adapted treatment approach. *Third*, we will include patients’ real-world treatment preferences and shared-decision making elements in the course of the experimental therapy.

The results of this randomized comparison of rTMS as first-line treatment and SSRI will allow for the data-informed design of a full-size clinical trial with the potential to substantially change MDD treatment by expanding the indication for rTMS to early phases of MDD, re-evaluating the positioning of rTMS in the course of therapy and documenting the feasibility and efficacy of participatory decision-making. An expansion of therapeutic options, particularly in early phases of the disorder, is highly desirable for preventing treatment resistance and reducing chronic courses. Not least, an extended range of therapeutic options can increase treatment satisfaction and success by integrating patient’s preferences in individualized treatment planning.

## Design and methods

**Fig. 1:**
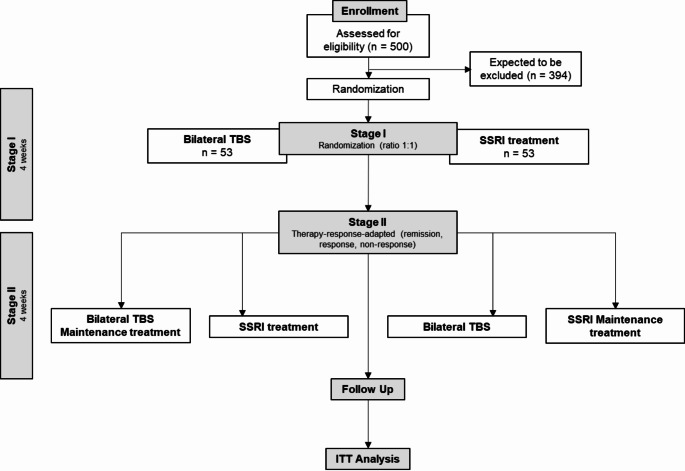
Trial flow

We will conduct a two-stage therapy response-adapted, rater-blinded, randomized (1:1 ratio) and controlled study (Fig. 1). Each stage will encompass a 4-week treatment. The follow-up phase covers a period of three months.

### Patient allocation to Stage I and Stage II and treatment modalities

For each patient the trial starts with obtaining written informed consent and checking the inclusion and exclusion criteria. In case of eligibility, randomization will determine whether a participant will receive one of two treatment approaches consistent with guideline-based clinical practice in ***Stage I*** (4 weeks) either 20 sessions of bilateral TBS (each weekday) or SSRI treatment (daily intake), respectively.

In ***Stage II*** (4 weeks), the allocation to the following treatment phase relies on the response to the treatment of Stage I. The decision will be made within the framework of a shared decision-making process. There are three different scenarios depending on the development of the Montgomery-Asberg Depression Rating Scale (MADRS) score after Stage I:

First, in the case of ***remission*** achieved after Stage I (remission is defined as MADRS score ≤ 10 points), patients will receive maintenance therapy i.e. maintaining the medication or continuing TBS in the sense of a TBS maintenance therapy (no TBS in week 1, 5 TBS sessions in week 2, no TBS in week 3, 5 TBS sessions in week 4 of Stage II). In this case, a shared decision-making process regarding further treatment is obsolete, as patients meet no longer the criteria for MDD.

Second, in case of ***non-response*** (MADRS score increase or reduction by < 50%), treatment condition will switch to the respective other arm. Continuation of ineffective treatment would be inconsistent with guideline-based clinical practice.

Third, in case of ***treatment response*** (MADRS score reduction by ≥ 50% compared to baseline), but not meeting remission criteria, maintenance treatment will be generally recommended. However, the patient’s individual preference will be considered and discussed. We will prepare a standardized and comprehensive patient information leaflet comprising comprehensible and detailed information regarding the mechanisms and (dis)advantages of the two treatment alternatives. Patients will thus be provided with information material that empowers them to make an informed decision on the individually preferred treatment condition in Stage II (maintenance or switch). We will document the patients’ individual reasons for their treatment preferences.

### Interventions

#### Bilateral TBS

In Stage I, bilateral TBS consists of 20 sessions administered daily from Monday to Friday over a period of 4 weeks. The target regions are the left and right dorsolateral prefrontal cortex (dlPFC), EEG position F3 and F4 respectively. Previous studies have shown a significant clinical improvement in depressive symptoms after a treatment duration of 4–6 weeks [23–25].

On the left hemisphere, iTBS is applied, which has a stimulating effect on the target region similar to 10 Hz rTMS [18, 19, 24]. On the right dlPFC, cTBS is applied, which has an inhibitory effect similar to 1 Hz rTMS [18, 19, 26]. Before and after each stimulation, a safety check is performed, including visual inspection of the target region as well as assessing and documenting the occurrence of (serious) adverse events.

### Stimulation localization, stimulation protocol, and technical devices

Based on the standard TBS protocols [27–29], each stimulation session consists of 600 biphasic stimuli applied in bursts of 3 pulses at 50 Hz, with an interstimulus interval of 200 ms. iTBS is administered 20 times for 2 s to the left dlPFC, with an intertrain interval of 8 s and cTBS continuously for 40 s to the right dlPFC in alternating order. A standard figure-8 coil will be used.

To individualize stimulation intensity, each patient’s resting motor threshold (RMT) is determined before the start of the first treatment session. RMT is defined as the minimum stimulus intensity that produces a minimal, visible motor response (in at least 5 of 10 trials) at rest. Stimulation intensity is set to 80% of the RMT for both iTBS and cTBS. The localization of the TBS target regions is based on the 10/20 EEG system. The left dlPFC corresponds to F3 and the right dlPFC to F4 [6, 30–32].

### Standard of care treatment with SSRI

SSRI are considered as a first-line treatment of at least moderate MDD [33]. Escitalopram (10 mg / day) will be administered as standard medication. In justified individual cases and according to the decision of the study physician, the prescription of a higher dose or another SSRI is possible. Before starting medication, a comprehensive physical examination, an electrocardiogram (ECG), and routine blood tests are performed to identify any potential contraindications that would exclude participants from the study. Each woman of childbearing age will carry out a pregnancy test. During treatment, participants are instructed to contact the study physician in cases of experiencing any adverse events (AEs). Adverse events/severe adverse events (AE/SAEs) are recorded on a daily basis on the designated documentation form.

### Study population

The study population comprises patients with at least moderately severe MDD. This can be either the first or a recurrent episode, but participants have to be medication-naïve according to the Antidepressant Treatment History File (ATHF) [34] for the whole history of MDD.

#### Inclusion criteria

Male, female, or diverse sex; age between 18 and 65 years; moderate to severe MDD according to the diagnostic criteria of DSM-5; MADRS Score ≥ 20 points; the duration of the episode must be at least 2 weeks and not exceed a period of 2 years; no previous antidepressant treatment according to the ATHF; indication for antidepressant medication; capability to give informed consent.

#### Exclusion criteria

Acute suicidal ideation (MADRS item 10 score > 4); presence of psychotic symptoms; antiepileptic drugs or benzodiazepines in a dosage equivalent to > 1 mg Lorazepam/d; comorbid axis I disorder (except anxiety disorders); presence of severe, clinically relevant, and predominant comorbid personality disorder; treatment resistance defined as the failure of at least one adequate antidepressant treatment attempt in the current or previous depressive episode; neurological pre-existing conditions such as severe traumatic brain injury, neoplasms, brain surgery, stroke within the last 3 months, neurodegenerative diseases: epilepsy or history of epileptic seizures; cardiac pacemaker (not compatible with MRI); intracranial metallic implants; previous rTMS treatment; deep brain stimulation; other severe somatic diseases; pregnancy; contraindications for the use of escitalopram.

### Ethics, consent and registration

Our study will be conducted in accordance with the principles of the Declaration of Helsinki and comply with the guidelines of Good Clinical Practice of the International Conference on Harmonization of Technical Requirements for Registration of Pharmaceuticals for Human Use. The Ethics Committee of the Medical Faculty of the University of Tübingen has approved the protocol, patient information, and consent form of this clinical trial (protocol version 1.1, approved on 03 July 2024, 614/2023BO1). All study participants will receive comprehensive information about the nature, scope, and procedures of the investigations and will give written informed consent prior to inclusion. Only patients who display sufficient capacity for insight, judgment, and consent (verified by qualified medical trial staff) will be included in the trial. In addition, all study participants will be informed of their right to discontinue participation in the study at any time without stating reasons and without disadvantages.

### Study centers and recruitment

Besides the Department of Psychiatry and Psychotherapy of the University Hospital Tübingen as the coordinating center, four additional German centers are planned to participate in the recruitment and treatment of study participants. Potential centers must meet the following requirements for participating: availability of the appropriate technical equipment and qualified personnel with experiences in the TMS treatment of depressive patients as well as the capacity to include at least 12 patients in the trial. The recruitment will take place in the outpatient departments of the participating centers. In addition, potential participants can be addressed by contacting local patient advocacy/support groups, local psychiatrists/general practitioners, as well as information via the respective clinic’s websites, social media, newsletters, local newspapers, and/or advertisement in public transportation.

### Patient involvement

The study was designed in cooperation with members of the German Center for Mental Health, (*Deutsches Zentrum für Psychische Gesundheit*, DZPG) stakeholder advisory board representing affected persons and relatives. Experts by Experience (EE) with lived experience regarding MDD are an integral part of our research team. They are regularly involved in the planning of the study, will support the trial until its completion and participate in the publication as well as dissemination of the results.

### Randomization

Randomization will take place after successful verification of the in- and exclusion criteria but before baseline measurements. The Institute of Clinical Epidemiology and Applied Biostatistics of the University of Tübingen (IKEaB) is responsible for randomization. By using the randomization tool of the software nQuery (release 8), a randomization list with varying block lengths was generated. According to this list the IKEaB created randomization envelopes with consecutive patient-ID. Each envelope contains the information whether a patient will start the treatment with either rTMS or the intake of escitalopram. Patients will be randomized by study staff not involved in the treatment.

### Sample size calculation

Two very recent studies comparing rTMS that incorporated a switch of antidepressants in patients with TRD have shown that rTMS is about 15–20% more efficient than the switch of medication [8, 9]. Considering this evidence and that only medication-naïve patients with MDD will be included, we expect a first stage response rate of 60% with rTMS and 40% with standard therapy (SSRI). Nevertheless, to ensure feasibility within the limitations of a phase II trial, even so assuming superiority, the study was powered only to show non-inferiority (with subsequent testing of superiority in case of non-inferiority). The non-inferiority criterion is set to -10% (i.e. an assumed 30% response rate). With a type one error of 0.025 (one-sided) and a statistical power of 80% we will need 84 evaluable patients. Considering 20% drop outs we want to recruit a total of 106 patients and randomize in a 1:1 ratio to both study arms.

### Statistical analysis

Statistical methods used to compare groups for primary and secondary outcomes:

We want to prove non-inferiority of early rTMS vs. standard SSRI. The primary analysis is carried out using a confidence interval of the difference between the two success rates at the end of Stage I. This interval is calculated using the normal distribution approximation for observed proportions using the formula (p 1 - p 2) +/- z * √ (p 1 (1 - p 1) / n 1 + p 2 (1 - p 2) / n 2), whereas p1 and p2 represent the observed frequencies of TMS respectively standard SSRI treatment represent; n1 and n2 represent the number of cases in these study arms (planned 42 evaluable per study arm). z means the 0.975 quantile of the standard normal distribution (1.96). Only this analysis is confirmatory. The null hypothesis “relevant inferiority of TMS” is rejected if that confidence interval only contains values ≥ -0.10. In this case, subsequently superiority of rTMS can be tested confirmatory (i.e. confidence interval only contains values ≥ 0). Methods for additional analyses, such as subgroup analyses and adjusted analyses:

Additional analyses are carried out using chi-square tests (categorical variables), and t-tests or Mann-Whitney tests depending on the scaling and distribution of the variables after Stage I (treatment comparison) or Stage II (sequence comparison). P-values are documented, but these are to be interpreted descriptively. Descriptive parameters are absolute and relative frequencies (categorical variables), mean values, standard deviations (normally distributed variables), medians and quartiles (other variables). The primary evaluation population is the Intention to Treat (ITT) population and includes all study participants who have started at least one treatment visit (i.e. at least 1 stimulation session or taking a single dose of SSRI). This population was chosen even so we perform a non-inferiority trial as we don’t expect change of study arms as reason for violating the Per Protocol (PP) criteria whereas non-adherence should be taken into account in the primary analysis. Missing primary endpoints are replaced using multiple imputation. No interim analysis will be performed. The software SPSS and/or R will be used for statistical analysis.

### Assessments

The screening consists of written informed consent, psychiatric examination including reviewing the inclusion- and exclusion criteria, documentation of sociodemographic data, physical and neurological examination, ATHF [34] and MADRS [35].

The baseline (R 0) comprises a pregnancy urine test (for women of childbearing age), determination of resting motor threshold (RMT), blood sampling, MADRS [35], Beck-Depression Inventory II (BDI-II) [36], patients’ preference regarding the treatment modality in Stage I, clinical global impression (severity scale) (CGI-S), World Health Organization Five Well-Being Index (WHO-5) [37], THINC-it Tool [38], Ruminative Response Scale-Short Form (RRS-short form) [39], General self-efficacy scale (GSE) [40], Rosenberg Self-Esteem Scale (RSES) [41], Credibility/ Expectancy Questionnaire (CEQ) [42], Childhood trauma questionnaire (CTQ) [43] and the Work Productivity and Activity Impairment Questionnaire (WPAI) [44].

During the treatment periods, the ratings R1-R4 will take place at the end of week two, four, six and eight. R2 (end of week 4) is the time point at which, among other ratings, the primary endpoint for this study is determined. R1 and R3 are interim evaluations only consisting of MADRS, BDI-II and CGI-I. R4 takes place at the end of Stage II. The follow-up rating R5 takes place after a three months period. The tests and questionnaires performed during Screening, Baseline, Ratings R1-R5 are summarized in Table 1.

With regard to the primary endpoint (MADRS) raters will be trained and blinded during the course of the study. The raters receive three videos explaining the ratings of the different MADRS items. A final video is used for evaluation, i.e. the question of whether the raters’ ratings correspond to the sample solution. Any discrepancies are discussed with the principal investigator and deviations of more than ± 1 point result in a repetition of the training. Based on these discussions, instructions for raters will be specified and communicated to all study centers.

**Table 1:** Tests, questionnaires and adjunctive measures (pregnancy test in urine, blood sampling, ECG and resting motor threshold) performed during S (screening), BL (baseline), the treatment period (stage I and II) and the Fup (follow up) are summarized. *only in cases of rTMS treatment, **only in cases of SSRI treatment

	S	BL	Stage I				Stage II				Fup
Week	0	0	1	2	3	4	5	6	7	8	20
Day	-10- -1	0		12		26		40		54	137
Rating Visits	S	R0		R1		R2		R3		R4	R5
Informed consent	X										
Inclusion and exclusion criteria	X										
Demographic data	X										
Neurological and physical examination	X										
ATHF	X										
Randomization		X									
Pregnancy test in urine		X									
Resting motor threshold		X*				X*					
Blood sampling		X**				X**					
ECG		X**				X**					
MADRS	X	X		X		X		X		X	X
BDI-II		X		X		X		X		X	X
CGI-S		X				X				X	
CGI-I				X		X		X		X	
WHO-5		X				X				X	X
Thinc-it tool		X				X				X	X
RRS-short form		X				X				X	
GSE		X				X				X	
RSES		X									
CEQ		X				X					
SDM-Q						X					
CTQ		X									
WPAI		X				X				X	
Safety check			*Will be performed continuously*								

### Study timeline

Figure 1 gives an overview of the study timeline. Following written informed consent participants who meet the in- and exclusion criteria will be randomized in Stage I either to 4 weeks of TBS or 4 weeks SSRI treatment. The allocation to Stage II depends on treatment response and patients’ preference. Patient participation will end with the follow-up rating after five months.

### Primary and secondary endpoints

The *primary endpoint* is the comparison of the two study arms with regard to therapy response (reduction of MARDS score ≥ 50%) after 4 weeks at the end of Stage I (R2).

*Secondary endpoints* are the comparison of the two study arms with regard to remission rates after 4 weeks at the end of Stage I (MARDS ≤ 10). Changes in the severity of depressive symptoms after Stage I and II in terms of differences in pre- and post-values of the MADRS and BDI-II as well as achievement of remission at the end of Stage II. The CGI-S/CGI-I to quantify the general clinical improvement. The WHO-5 to capture each patient’s assessment of change in his or her mental well-being [37]. Further secondary endpoints are changes in cognitive functioning measured by the THINC-it Tool [38], incidence of AEs/SAEs in the respective treatment stages and arms as well as dropout rates in the different treatment arms. Further instruments will provide information about mechanisms and potential predictive markers of treatment response: RRS-short form [39], CTQ, GSE [40], RSES [41], CEQ [42], and if applicable, the Shared decision-making questionnaire (SDM-Q) [45]. The WPAI is used to assess the impact of health conditions on work productivity and daily activities.

### Monitoring and data reviewing

Study sites will entry the data in an electronic clinical record file (eCRF) by using the software REDCap. The IKEaB will regularly inspect the database entries for completeness and plausibility by performing query runs. In the course of programming the eCRFs, plausibility checks and mandatory fields (for particularly critical items, e.g. primary outcome) will already be included in order to limit potentially incorrect or missing entries. The leading study site will be informed if irregularities or missing data occur. All sites will receive training on how to use the eCRFs. Clinical study management and IKEaB will be responsible for query management and data cleaning.

## Discussion

This study will provide first evidence for the efficacy of TBS as first-line treatment in medication-naïve MDD patients compared to antidepressant medication. By using a two-stage, therapy response-adapted study design we also investigate the relevance of intervention order as well as the role of patients’ treatment preferences.

Pharmacological and psychotherapeutic treatment are well studied in MDD and have been proven to be effective [46]. Nevertheless, a significant proportion of patients do not respond adequately to therapy [47]. The occurrence of side effects [48] and difficulties in discontinuing medication [49] are further disadvantages leading to a reduction in patients’ acceptance and tolerance. Additionally, complex treatment situations and certain patient groups, such as pregnant women or patients with severe somatic illnesses or polypharmacy, for whom pharmacological options are per se limited, highlight the need for alternative treatment strategies as well as the fact that psychotherapeutic treatment options are only available for a limited proportion of patients.

The clinical efficacy and benefits of rTMS are widely accepted [50], yet available rTMS studies in MDD focus almost exclusively on evaluating its effectiveness in treating varying degrees of treatment resistance. To date, there is a research gap regarding the comparison of rTMS with other treatment options such as antidepressants in general and in particular regarding forms of MDD, which respond inadequately to antidepressants. However, very recently, rTMS was compared to a Dutch antidepressant medication algorithm with switching or augmentation strategies in 89 patients with TRD resulting in a 20% higher response rate in the rTMS group [8]. In another study, 278 patients with TRD received either rTMS, aripiprazole augmentation or switch to serotonin and norepinephrine reuptake inhibitors (SNRI). Here, rTMS but not aripiprazole was shown to be superior to the switch of the antidepressant [9].

However, the current evidence supporting the use of rTMS as an early intervention, particularly when compared to standard antidepressant treatment, is still scarce. This lack of high-quality randomized controlled trials hampers the translation of rTMS as an early intervention into clinical practice. We found only two systematic reviews [51, 52]. In the first review, Zheng et al. (2023) summarize the effects of rTMS in children and adolescents [51]. Three of the mentioned studies included medication-naïve MDD patients, but no study compared the effects of TBS with antidepressant medication. In the second review, Voigt et al. (2019) give an overview about the available research regarding rTMS as an early intervention in MDD. Two randomized controlled trials (RCTs) included patients with first-episode MDD, but investigated rTMS only as an add on treatment to an initiated pharmacotherapy [14, 15]. Huang et al. (2012) investigated the effectiveness of two weeks active 10 Hz rTMS as an add on to citalopram compared to sham rTMS to the left dlPFC. They observed a greater number of early improvers with regard to depressive symptoms in the active rTMS group. However, there was no significant difference regarding therapy response and/or remission rates after 4 weeks of treatment [14]. Wang et al. (2017) also examined first-episode MDD patients who received 4 weeks of active 10 Hz rTMS to the left dlPFC in combination with paroxetine compared to an add on sham stimulation. After the 4 weeks rTMS interval, the administration of paroxetine was continued for further 4 weeks. In line with the results mentioned above, the active rTMS group showed a significantly faster improvement of depressive symptoms. However, there was no significant difference regarding therapy response and remission at the end of the study [15]. These results suggest that rTMS as an add-on therapy can accelerate the onset of symptom improvement in first-episode depression. However, the duration of rTMS was relatively short and the efficacy compared to standard SSRI treatment remains unknown. One recent systematic review and meta-analysis showed that rTMS significantly reduced suicidal ideation in medication-naïve MDD patients [53], whereas one of the included studies investigated depression after traumatic brain injury including neuropsychiatric symptoms [54] and the other cited study also compared active versus sham rTMS as an add on to escitalopram [55]. Furthermore, we identified one ongoing trial, which aims to investigate the effect of iTBS in medication-naive MDD patients compared to the effect in TRD (ID NCT04000022).

To summarize, our systematic literature research indicated that there is no RCT comparing TBS as an early intervention in adult medication-naïve patients with pharmacotherapy.

To fill this gap, we decided to conduct a two-stage, therapy response-adapted study design with special consideration of the patients’ preference within the framework of a shared decision-making process after Stage I of the study. The design is inspired by the methodological principles of the SMART-design (Sequential Multiple Assignment Randomized Trial) to evaluate the efficacy of different treatment regimens over time. With this approach, it is possible not only to compare the efficacy of two treatment options, but also to evaluate which sequence is most beneficial [56]. Unlike a cross-over trial, this study allows to compare two treatment sequences, with the second treatment option only being used if the first fails. The results will deliver the essential basis for a future confirmatory full-size response-adapted clinical trial.

An important limitation of this study is that no conclusions can be drawn about the full efficacy potential of rTMS compared to SSRIs in non-TRD patients. This would require a substantial prolongation of Stage I for up to 12 weeks. However, continuing treatment (rTMS or SSRI) for more than 4 weeks in this particular patient group is contrary to (German) treatment guidelines and would therefore not be approved by the ethics committee.The efficacy of rTMS has already been proven in TRD. Since it is not plausible that lower levels of treatment resistance show lesser response to rTMS and the individual expectation of treatment efficacy has an important and clinically highly relevant influence on effectivity [22], we decided to compare the real-world clinical use of the two treatment options without a placebo control.

If our study will yield positive results, this will have significant implications for expanding the range of available first-line treatment options for MDD, allowing for more tailored and individualized treatment plans. By including patients’ preference in the treatment decision process, we acknowledge patients’ real-world autonomy leading to clinically more meaningful results. Not least, involving patients in treatment decisions can activate positive expectations towards efficacy and tolerability and potentially result in better outcomes [57]. TBS as an additional first-line treatment option in early phases of MDD may help to enhance treatment effectivity, improve patient satisfaction and thus expand the options to act against treatment resistance in MDD.
